# Influence of age on retinochoroidal healing processes after argon photocoagulation in C57bl/6j mice

**Published:** 2009-04-03

**Authors:** C. Dot, V. Parier, F. Behar-Cohen, D. BenEzra, L. Jonet, B. Goldenberg, E. Picard, S. Camelo, Y. de Kozak, F. May, G. Soubrane, J. C. Jeanny

**Affiliations:** 1 Centre de Recherche des Cordeliers, Université Pierre et Marie Curie, Paris, France; 2 Université Paris Descartes, UMR S 872, Paris, France; 3 INSERM, U872, Paris, France; 4 Service d’Ophtalmologie, Hôpital d’Instruction des Armées Legouest, Metz, France; 5 Service d’Ophtalmologie, Hôpital Intercommunal de Créteil, France; 6 Service d’Ophtalmologie, Hotel Dieu, Paris, France; 7 Assuta Medical Centre-Rishon, Israel

## Abstract

**Purpose:**

To analyze the influence of age on retinochoroidal wound healing processes and on glial growth factor and cytokine mRNA expression profiles observed after argon laser photocoagulation.

**Methods:**

A cellular and morphometric study was performed that used 44 C57Bl/6J mice: 4-week-old mice (group I, n=8), 6-week-old mice (group II, n=8), 10–12-week-old mice (group III, n=14), and 1-year-old mice (group IV, n=14). All mice in these groups underwent a standard argon laser photocoagulation (50 µm, 400 mW, 0.05 s). Two separated lesions were created in each retina using a slit lamp delivery system. At 1, 3, 7, 14, 60 days, and 4 months after photocoagulation, mice from each of the four groups were sacrificed by carbon dioxide inhalation. Groups III and IV were also studied at 6, 7, and 8 months after photocoagulation. At each time point the enucleated eyes were either mounted in Tissue Tek (OCT), snap frozen and processed for immunohistochemistry or either flat mounted (left eyes of groups III and IV). To determine, by RT–PCR, the time course of glial fibrillary acidic protein (*GFAP*), vascular endothelial growth factor (*VEGF*), and monocyte chemotactic protein-1 (*MCP-1*) gene expression, we delivered ten laser burns (50 µm, 400 mW, 0.05 s) to each retina in 10–12-week-old mice (group III’, n=10) and 1-year-old mice (group IV’, n=10). Animals from Groups III’ and IV’ had the same age than those from Groups III and IV, but they received ten laser impacts in each eye and served for the molecular analysis. Mice from Groups III and IV received only two laser impacts per eye and served for the cellular and morphologic study. Retinal and choroidal tissues from these treated mice were collected at 16 h, and 1, 2, 3, and 7 days after photocoagulation. Two mice of each group did not receive photocoagulation and were used as controls.

**Results:**

In the cellular and morphologic study, the resultant retinal pigment epithelium interruption expanse was significantly different between the four groups. It was more concise and smaller in the oldest group IV (112.1 µm±11.4 versus 219.1 µm±12.2 in group III) p<0.0001 between groups III and IV. By contrast, while choroidal neovascularization (CNV) was mild and not readily identifiable in group I, at all time points studied, CNV was more prominent in the (1-year-old mice) Group IV than in the other groups. For instance, up to 14 days after photocoagulation, CNV reaction was statistically larger in group IV than in group III ((p=0.0049 between groups III and IV on slide sections and p<0.0001 between the same groups on flat mounts). Moreover, four months after photocoagulation, the CNV area (on slide sections) was 1,282 µm^2^±90 for group III and 2,999 µm^2^±115 for group IV (p<0.0001 between groups III and IV). Accordingly, *GFAP*, *VEGF*, and *MCP-1* mRNA expression profiles, determined by RT–PCR at 16 h, 1, 2, 3, and 7 days postphotocoagulation, were modified with aging. In 1-year-old mice (group IV), *GFAP* mRNA expression was already significantly higher than in the younger (10–12 week) group III before photocoagulation. After laser burns, *GFAP* mRNA expression peaked at 16–24 h and on day 7, decreasing thereafter. *VEGF* mRNA expression was markedly increased after photocoagulation in old mice eyes, reaching 2.7 times its basal level at day 3, while it was only slightly increased in young mice (1.3 times its level in untreated young mice 3 days postphotocoagulation). At all time points after photocoagulation, *MCP-1* mRNA expression was elevated in old mice, reaching high levels of expression at 16 h and day 3 respectively.

**Conclusions:**

Our results were based on the study of four different age groups and included not only data from morphological observations but also from a molecular analysis of the various alterations of cytokine signaling and expression. One-year-old mice demonstrated more extensive CNV formation and a slower pace of regression after laser photocoagulation than younger mice. These were accompanied by differences in growth factors and cytokine expression profiles indicate that aging is a factor that aggravates CNV. The above results may provide some insight into possible therapeutic strategies in the future.

## Introduction

It is estimated that age-related macular degeneration (AMD) affects 12–15 million Americans and around 1 million French over the age of 65. This disease is complicated in 10%–15% by the occurence of choroidal neovascularization (CNV) causing visual loss [1,2]. CNV formation is associated with vision loss even when the new vessels are not yet “active” (no exudation). [2]. Spontaneous regression of the CNV is rarely observed in AMD, while continuous growth and steady deterioration of vision is characteristic. These lesions may often progress to form large fibrovascular scars [3,4]. CNV associated with AMD has a extended surface than CNV in other diseases occurring mostly in younger age groups of patients (high myopia, angioid streaks, chronic choroidopathies) [2,5]. These observations suggest that aging in human predisposes to the formation of more severe CNV formation without evident self limitation or regression [3].

The purpose of the present study was to examine the role of age as an independent factor determining the severity of CNV formation and its long-term evolution after argon laser photocoagulation (PC) in the mouse eye. The CNV analysis was performed by two different strategies: Morphometry on histological sections or flat mounts and molecular expression of genes implicated in angiogenesis, inflammation and glial activation.

## Methods

### Animals

The present study used 64 C57Bl/6J mice of different ages (Janvier, Le Genest-Saint-Isle, France), which were treated and analyzed. Four untreated mice were also included as controls. For the morphologic and cellular study, four groups of mice were used. At the time of laser photocoagulation, their weight and age were as follows: Group I, 4-week-old mice (very young mice, weighing 12–13 g; n=8); Group II, 6-week-old mice (young mice, weighing 15–18 g; n=8); Group III, 10–12-week-old mice (young adult mice, weighing 17–21 g; n=14); Group IV, 12-month-old mice (old mice, weighing 25–28 g; n=14). For the study of mRNA expressions, RT–PCRs for glial fibrillary acidic protein (*GFAP*), vascular endothelial growth factor (*VEGF*), and monocyte chemotactic protein-1 (*MCP-1*) were performed in mice from group III’ (n=10) and from group IV’ (n=10).

Mice were fed a standard laboratory diet and given tap water ad libitum. They were maintained on a 12 h:12 h light-dark cycle in a temperature-controlled room at 21–23 °C. Before treatment, mice were anesthetized by intraperitoneal injection of 0.15 ml (40 mg/kg) of sodium pentobarbital (Sanofi Santé Animale, Libourne, France), diluted 1:10 in balanced salt solution (BSS). Their pupils were dilated with 2 mg/0.4 ml 1% tropicamide (Tropicamide Faure, Novartis Pharma, Rueil-Malmaison, France). All experimental procedures were performed in accordance with the Association for Research in Vision and Ophthalmology (ARVO) statement for the use of animals in ophthalmic and vision research.

### Laser photocoagulation

Quantel medical model argon laser photocoagulator (Viridis 532nm, Quantel Medical, Clermont-Ferrand, France) mounted on a slit lamp (Hagg-Streitt, BQ 900) was used throughout. A glass coverslip fulfilled the role of a contact lens during the laser delivery. In the cellular and morphologic study, two photocoagulation lesions (50 µm spot size, 0.05 s duration and 400 mW power) around the optic nerve, 1 to 2 disc diameters away from the papillae, were created in both eyes of all experimental mice. The relative laser burn intensity was calculated as 0.40. In all treated eyes included in the study, a reactive bubble at the retinal surface was observed after laser delivery. This was considered evidence for appropriate focusing and as an indication of the rupture of Bruch’s membrane [6-8].

In the molecular study, ten laser spots (50 µm, 0.05 s, 400 mW) were uniformly realized on the total surface of the retina. These spots were performed at a distance of 1–2 disc diameters from the optic nerve.

### Postphotocoagulation treatment

In the cellular and morphologic study, two animals from each group were sacrificed by carbon dioxide inhalation at day 1, 3, 7, and 14, as well as 2 and 4 months after photocoagulation. Mice were also sacrificed at 6, 7, and 8 months for groups III and IV. Eyes were enucleated immediately after euthanasia and mounted in Tissue Tek (OCT; Bayer Diagnostics, Puteaux, France), snap frozen, cut, and processed for immunohistochemistry or used for flat mount preparations of the neural retina and retinal pigment epithelium (RPE)-choroid-sclera complex.

For the molecular study, the neural retina and RPE-choroid complex of two animals from each group (III’ and IV’) were collected at 16 h, 1, 2, 3, and 7 days after laser photocoagulation. RNA was isolated and used for RT–PCR. Two untreated mice from each group age (without photocoagulation) were used as controls.

### Immunohistochemistry

From eyes mounted in OCT, serial frozen sections 10 µm thick were obtained using a Leica CM3050S cryostat (Leica, Rueil-Malmaison, France). After localization of the laser beam, all sections 100 µm before the lesion, throughout the lesion, and 100 µm beyond it were systematically collected and further processed. Transversal changes within the same laser impact as well as longitudinal changes with elapsing time after photocoagulation were analyzed.

Cryosections were collected on glass slides and fixed in 4% paraformaldehyde (PAF; LADD, Inland Europe, Conflans-sur-Lanterne, France) for 5 min at room temperature. Specific polyclonal rabbit antibodies against GFAP (Dakocytomation, Trappes, France) were used for the detection of astrocytes and activated Müller cells and specific polyclonal rabbit antibodies against vW factor (Dakocytomation) were used for the detection of vascular endothelial cells. The slides were incubated 1 h at 20–22 °C with the first primary antibody diluted 1:100 in phosphate buffer saline (PBS without CaCl_2_ and Mg Cl_2_, Gibco distributed by Invitrogen, Cergy Pontoise, France). After washing, sections were incubated for 60 min in a solution of 1:100 secondary goat anti-rabbit antibodies conjugated to Alexa 488 (Molecular Probes, Interchim, Asnières, France). After these first incubations, sections were incubated successively with the second primary antibody and donkey anti-rabbit antibodies conjugated to Texas Red (Jackson Immuno Research, Interchim, Montluçon, France), both diluted 1:100 in PBS. The slides were then washed, stained 5 min with 4’,6-Diamidino-2-Phenyl-Indole (DAPI; Sigma-Aldrich, Saint Quentin Fallavier, France) diluted 1:3,000, washed again in PBS, then mounted in 1:1 glycerol-PBS. Stained sections were observed under a fluorescence microscope Aristoplan (Leica) and photographed with a Spot RT digital camera (Optilas, Evry, France) with a 25X objective. Negative controls were cryosections reacted with nonimmune serum or cryosections in which the primary specific antibody was omitted. Each staining was performed on a minimum of 15 independent sections. Analysis of the cellular remodeling processes taking place within the laser lesion and 100 µm around it was systematically performed for each time point.

### Flat mount preparation

Flat mounts of the neuroretina and of the remaining RPE-choroid-sclera complex were prepared. The enucleated eyes were incised at the limbus and immediately fixed for 15 min with paraformaldehyde (PAF) 4% solution in PBS. These were washd three times with PBS, and the anterior segments were dissected out. The neuroretina was then carefully separated from the RPE-choroid-sclera complex. Post-fixation with methanol 100% for 15 min at −20 °C was performed, and neuroretina and RPE-choroid-sclera complex further processed as follows: rehydratation with PBS plus 1% Triton X-100 and incubation overnight with lectin from *Bandeiraea simplicifolia* conjugated to fluorescein isothiocyanate (Lectin, FITC labeled, from Bandeiraea simplicifolia, BS-I, Ref. L9381, Sigma Aldrich) and a primary antibody against GFAP, both diluted 1:100 in PBS-Triton 0.1%. After this first incubation, the samples were washed with PBS and incubated for 1 h with a goat anti-rabbit IgG coupled to Texas Red diluted 1:100. After this last incubation, the neural retina and the remaining choroid complex were washed thoroughly with PBS and flat mounted between a slide and a coverslip using gel mount (Biomeda Corp., VWR, Fontenay-sous-Bois, France). Flat mounts were observed under an Aristoplan fluorescence microscope (Leica) and photographed with a spot RT digital camera (Optilas) with a 25X objective.

### Statistical analysis

Surface area of CNV was determined by using either lectin from *Bandeiraea simplicifolia* fluorescence (flat mounts) or vW factor fluorescence (serial sections), and outlining the margins of the lesion with the image-analysis software (Visilog 6.2. Noesis, Les Ulis, Courtaboeuf, France). The areas were calculated in µm^2^ after calibration of the software. Each time point was analyzed on a minimum of three independent samples (flat mounts) or 15 serial sections within the laser beam center. Phase contrast images were used for the other morphometric analysis. The size of the RPE interruption was calculated with the image-analysis software (Visilog 6.2).

The mean and standard deviation (SD) for all measures of each time point was calculated and statistics (*t*-test) were determined by computer (Prism 4.0, Graph Pad Software Inc., San Diego, CA). Results at p<0.05 were considered to be statistically significant for all forms of statistical analyses used.

### Assessment of mRNA Levels by RT–PCR

Mice were sacrificed by carbon dioxide inhalation. Their eyes were enucleated, anterior segments removed, and neuroretina and RPE-choroid-sclera complex dissected. Total RNA was isolated from both tissues by the acid guanidinium thiocyanate-phenol-chloroform method [9]. For their potential role in angiogenesis, expression of mRNAs for *GFAP*, *VEGF*, and *MCP-1* was analyzed. Sense and antisense primers for *18S* and *VEGF* were obtained from MWG-Biotech AG (Paris, France), for *GFAP* from Sigma-Proligo (St. Quentin Fallavier, France), for *MCP-1* from Sigma-Aldrich (Paris, France). Table 1 lists the sequences used in this study. Primers were designed to amplify specifically the cDNA fragments representing mature mRNA transcripts of 70 bp for *18S*, 84 bp for *GFAP*, 202 bp for *VEGF*, and 274 bp for *MCP-1*. The PCR fragments were analyzed by 3% agarose gel electrophoresis and visualized by ethidium bromide staining under UV. Mouse *18S* primers were used to provide an internal control for the amount of template in the PCR reactions. The quantities of different mRNA present were expressed relative to the quantity of this *18S* housekeeping gene. The relative band intensity was calculated in comparison with *18S*, using the NIH Image 1.57 software package (Bethesda, MD).

**Table 1:** Overview of all primers used in the reverse transcriptase polymerase chain reaction.

**Gene**	**Sense primer**	**Antisense primer**
*18s*	AAGTCCCTGCCGTTTGTACACA	GATCCGAGGGCCTCACTAAAC
*GFAP*	ACCGCATCACCATTCCTGTAC	TGGCCTTCTGACACGATTT
*VEGF*	GTGAGCCAGGCTGCAGGAAG	GAATGC GTCTGCGGAGTCT
*MCP-1*	ACTGAAGCCAGCTCTCTCTTCCTC	TCCTTCTTGGGGTCAGCACAGAC

Sense and Antisense primers sequences used to detect mouse *18S, GFAP, VEGF,* and *MCP-1* genes with the reverse transcriptase polymerase chain reaction method.

## Results

Comparison of healing processes after argon photocoagulation between the four groups revealed similar cellular reactions. However, there were differences in the timing of appearance of the various cell types and their intensity.

**Figure 1:**
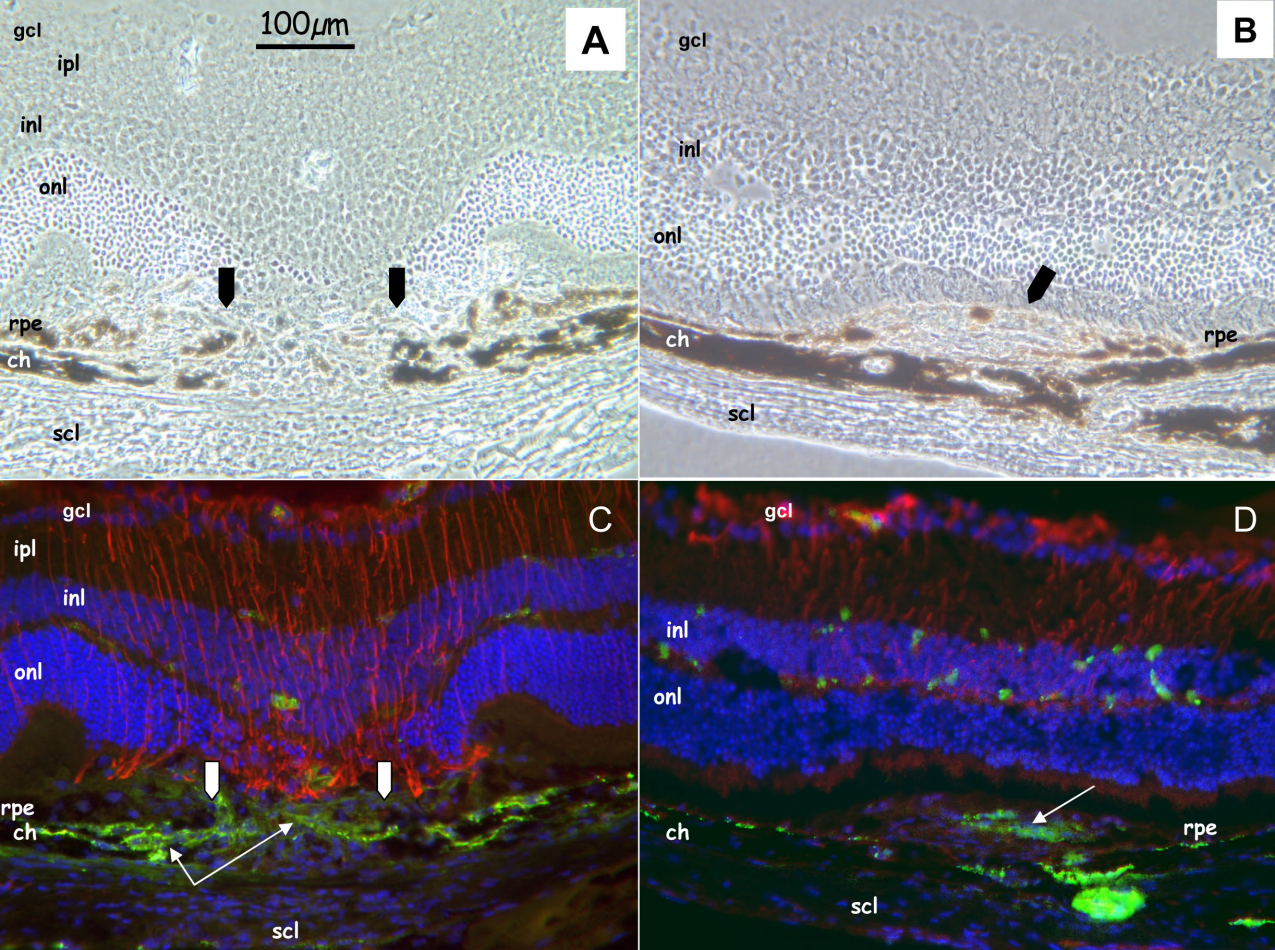
Cellular reactions after photocoagulation in 10–12-week-old mice (group III). Representative phase contrast images and immunohistochemistry of sections from eyes at day 7 (**A, C**), and month 6 (**B, D**) following photocoagulation. **A:** Phase contrast image demonstrates a localized disruption of retinal pigmented epithelium (rpe) and choroid (ch) in the center of the laser beam (arrows). **B:** Phase contrast image demonstrates the formation of a mound of poorly defined cells surrounded by lightly pigmented retinal pigmented epithelium (rpe) structures (arrow). **C:** Immunohistofluorescence analysis was performed with specific antibodies for von Willebrand (green) and glial fibrillary acidic protein (GFAP, red). Nuclei were counterstained with 4’,6-Diamidino-2-Phenyl-Indole (DAPI, blue). This section also illustrates a marked activation of GFAP-positive cells (astrocytes and Müller cells) in the center of the laser beam and von Willebrand reaction in the choroidal site (thin arrows). **D:** Six months after photocoagulation, a localized von Willebrand reaction is still evident at the level of the choroid (thin arrows). Abbreviations: ganglion cell layer (gcl), inner nuclear layer (inl), inner plexiform layer (ipl), outer nuclear layer (onl), sclera (scl). Scale bar (**A)** represents 100 µm for each panel.

In the four groups, serial sectioning revealed a localized disruption of the RPE cell layer and Bruch’s membrane, which demarcated well the lesion site (Figure 1A,B). On the retinal side, intense GFAP staining was observed from day 1 and was associated with activated retinal astrocytes and Müller cells, mostly in the axis of the laser beam. Müller cells dip toward the ruptured RPE cell layer “sealing” the disrupted choroid. Around the third day, vW-positive cells were observed at the external edges of the laser lesion. From day 7 onwards, these cells are more numerous at the vicinity of the disrupted RPE and Bruch’s membrane (Figure 1C). Due to the invagination of the GFAP-positive cells toward the impact center, these vW-positive cells were concentrated around the lesion edges. A thickening of the choroid lesion site was also detected along with a bulging of some RPE cells. Despite the continuous presence of CNV, the number of vW-positive cells decreased with time and were replaced by fibroblastic tissue surrounded by pigmented cells (Figure 1B,D).

**Figure 2:**
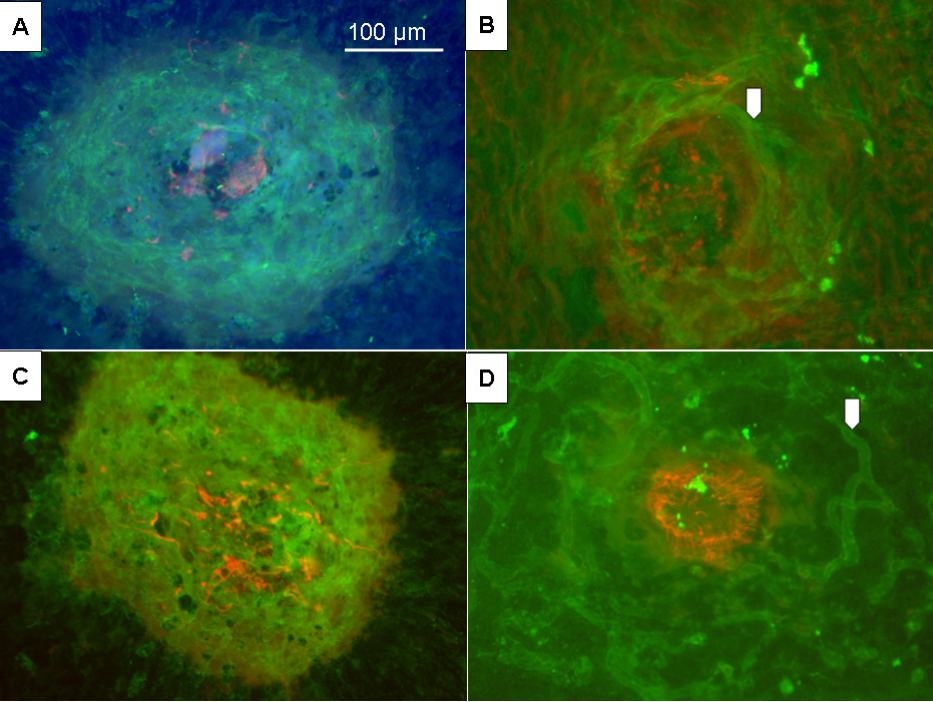
: Flat mounts showing choroidal neovascularization in mice from groups III and IV. Images of flat mounts of 10–12-week-old mice (group III; **A, B**) and 1-year-old mice (group IV; **C, D**), after immunolabeling done with lectin from Bandeiraea simplicifolia fluorescein isothiocyanate (FITC)-labeled (green), anti-glial fibrillary acidic protein (GFAP) antibodies (red) and 4’,6-Diamidino-2-Phenyl-Indole (DAPI; blue). **A:** At day 7 after photocoagulation (PC), new vessels surround the GFAP+cells activated in the center of the laser beam. The size of the neovascular reaction measures 399 µm in its largest diameter (initial laser beam of 50 µm). **B:** Six months after PC, new vessels are more delineated (arrow) around the activated GFAP+cells. **C:** At day 7 after PC, new vascular reaction extends like a pool. Size of the largest diameter measures 421 µm. **D:** Eight months after PC, Müller cells are still activated in the center of the laser beam. New vessels are dilated and appear fully developed (arrow). Scale bar (**A)** represents 100 µm for each panel.

In group III and IV mice, flat mount analysis demonstrated that the new vessels surrounded the activated GFAP + cells, localized within the center of the laser lesion. With elapsing time the new vessels matured and grew larger, surrounding nonvascular areas. Only occasionally, the new vessels were observed within the center of the laser beam (Figure 2).

The major differences in the behavior of the wound healing processes between the four age groups were the following: the resultant RPE interruption after photocoagulation was significantly smaller in the 1 year-old mice (group IV).

**Figure 3:**
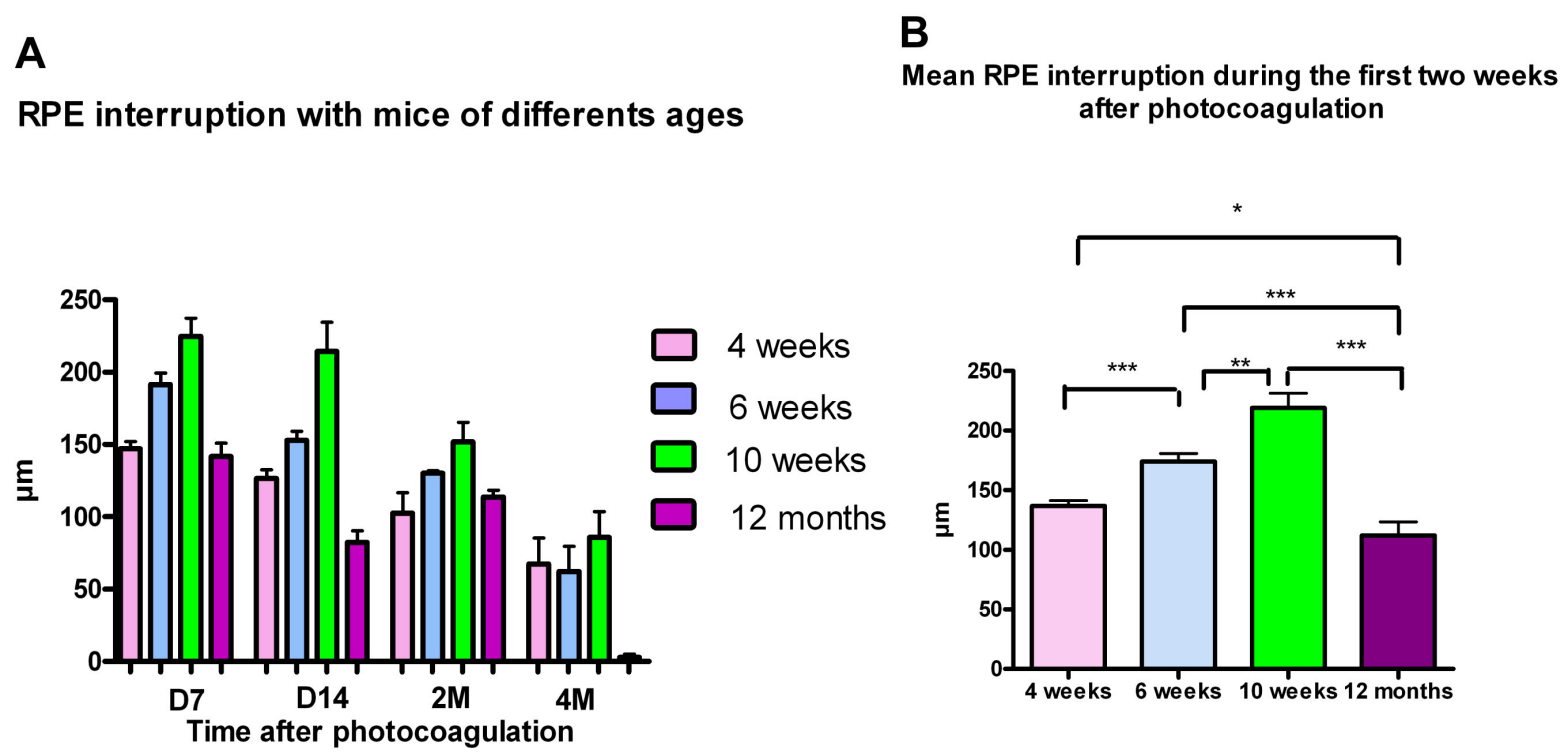
Size of RPE interruption after photocoagulation. Size of RPE interruption was evaluated for mice from the four groups (group I: 4 weeks, group II: 6 weeks, group III: 10–12 weeks, group IV: 12 months), from day 7 to month 4 after photocoagulation. **A:** The size of retinal pigment epithelium (RPE) interruption is maximal on day 7 after the laser burn in the four groups and decreases thereafter. It is larger in the 10–12-week-old mice (in green) at each time point. From month 4 a repair of the RPE layer is evident in 1-year-old mice; no interruption is recorded. **B:** The mean RPE interruption during the first two weeks after photocoagulation was as follows: 136.9 µm±4.4 in group I, 174.1 µm±6.6 in group II, 219.1 µm±12.2 in group III, and 112.1 µm±11.4 in group IV. The differences observed in the RPE interruption between mice from groups I, II III and mice from group IV are significant (p<0,0001) according to the *t*-test.

The mean RPE interruption (during the first two weeks after photocoagulation) was 136.9 µm±4.4 in group I, 171.1 µm±6.7 in group II, 219.1 µm±12.2 in group III. Interestingly, the RPE interruption caused in the oldest mice (group IV) was only 112.2 µm±11.4 (p<0.0001 between groups III and IV; p<0.0001 between groups II and IV; p=0.021 between groups I and IV) (Figure 3B). From month 4 onward, the RPE interruption in the older group was less clearly delineated with an evident RPE repair taking place. In the other groups, the RPE repair process and “filling of the gap” was slower (Figure 3A).

**Figure 4:**
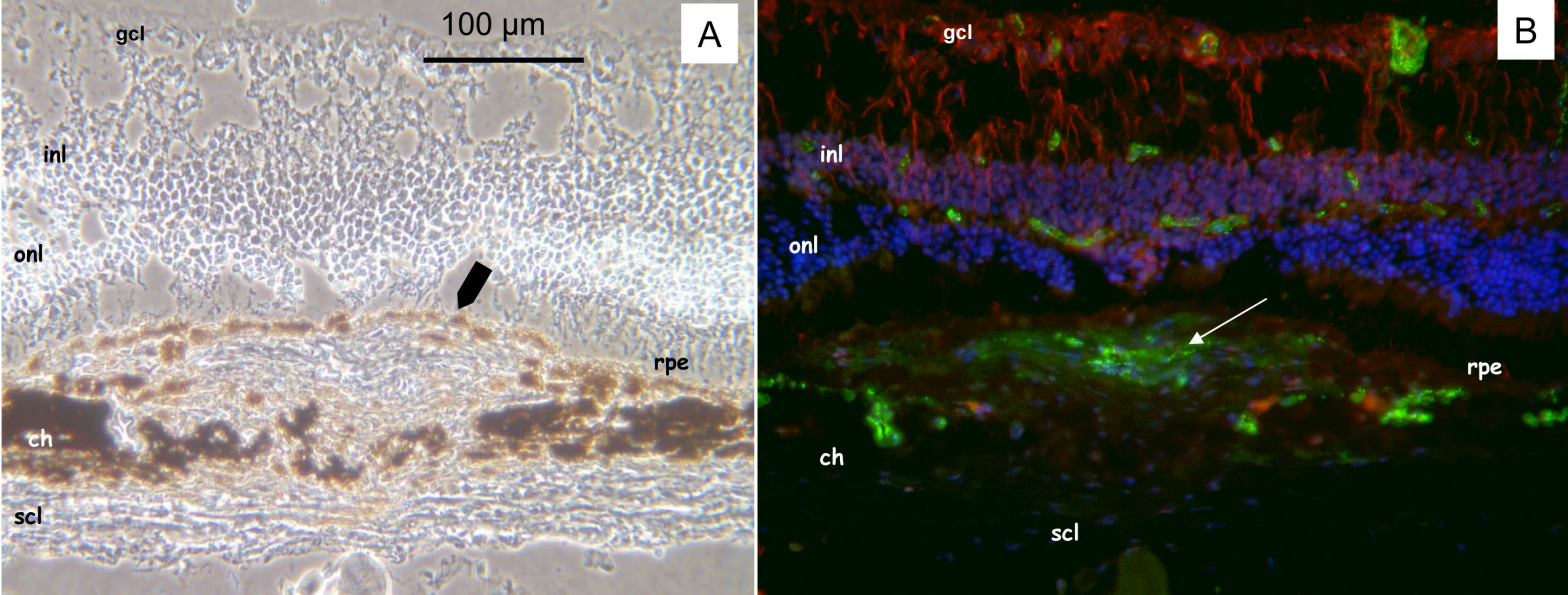
Representative phase contrast image and immunohistochemistry four months after photocoagulation in 1-year-old mice (group IV). **A:** Phase contrast image demonstrates a capsular and pigmented cell reaction that closes the initial retinal pigment epithelium (rpe) interruption and surrounds the choroidal neovascularization (CNV) reaction. Overlying the lesion, photoreceptor outer segments are markedly affected. **B:** Immunodetection of von Willebrand factor (green) reflects the CNV presence at least four months after photocoagulation limited by the surrounding new RPE. The new vessels do not infiltrate the retina. Astrocytes and activated Müller cells were detected with specific antibodies for glial fibrillary acidic protein (GFAP, red). Nuclei were counterstained with 4’,6-Diamidino-2-Phenyl-Indole (DAPI, blue). Scale bar in **A** represents 100 µm in both panels. Abbreviations: choroid (ch), ganglion cell layer (gcl), inner nuclear layer (inl), outer nuclear layer (onl), rod outer segments (ros), retinal pigment epithelium (rpe), sclera (scl).

**Figure 5:**
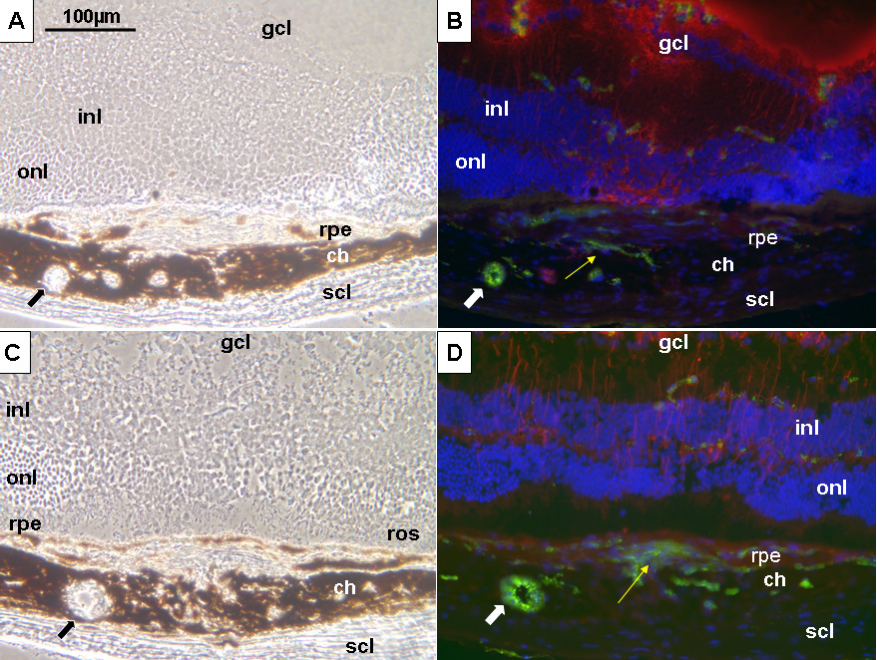
Cellular reactions after photocoagulation in young and old mice. Representative phase contrast images (**A, C**) and immunohistochemistry (**B, D**) at month 7 after photocoagulation in 10–12-week-old mice (group III; **A, B**) and 1-year-old mice (group IV) (**C, D**). **A, C:** Phase contrast images show a thickened choroid with pseudocystic cavities (black arrows). **B, D:** Immunofluorescence analysis was performed in 10–12-week-old mice (**B**) and 1-year-old mice (**D**). Choroidal neovascularization reaction is more prominent with aging (yellow arrows). Immunohistochemistry demonstrates that some of the pseudocystic cavities are vessels (white arrows). Scale bar (**A-D**) represents 100 µm. Abbreviations: choroid (ch), ganglion cell layer (gcl), inner nuclear layer (inl), outer nuclear layer (onl), rod outer segments (ros), retinal pigment epithelium (rpe), sclera (scl).

Phase contrast images demonstrated that RPE-like cells surrounded the CNV reaction (Figure 4). In the choroid underlying the laser lesion, pseudocystic cavities were observed. Immunohistochemistry of this site demonstrated that a few of these stained positively for von Willebrand and had choroidal vessels characteristics (Figure 5).

### CNV reaction was more extensive in the 1-year-old mice (group IV)

**Figure 6:**
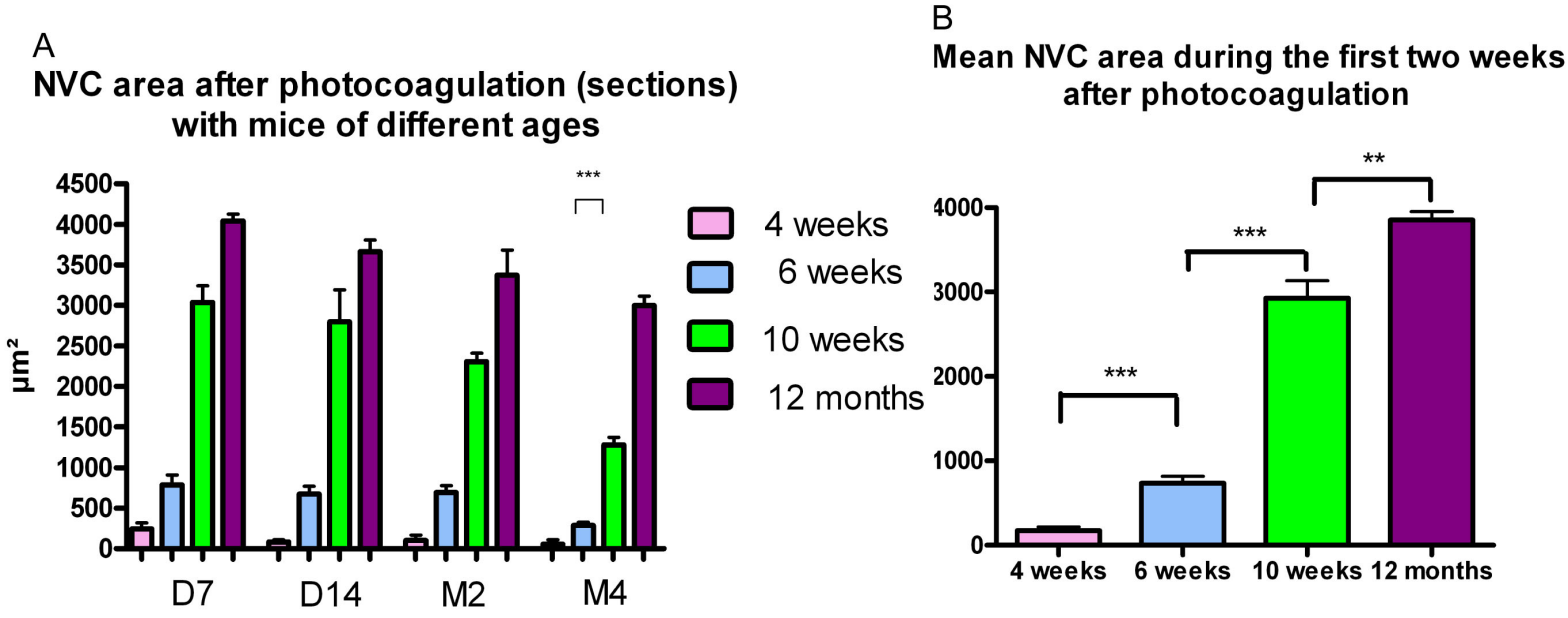
Evolution of the CNV reaction with aging. **A:** This reaction reaches its peak during the first two weeks after photocoagulation (PC), diminishes progressively after, and is still present on month 4. Choroidal neovascularization is rare and difficult to quantify in 4-week-old mice (group I). It is more prominent in 10–12-week-old mice and 1-year-old mice. It diminishes more markedly in young adult mice after two months. On month 4, the difference is statistically significant between young adult and old mice. A p<0.0001 was considered significant. **B:** The mean CNV area during the first two weeks after PC is significantly larger in old mice in comparison to young adult mice according to *t*-test.

On serial sections, vW-positive cells in the choroid around the lesion (CNV formation) were detected in the four groups of mice already three days after photocoagulation. These vW-positive cells were more evident from day 7 onward, reaching a peak on the second week after photocoagulation. In 4-week-old mice, a few mice present new vessels after photocoagulation, but the great majority of them do not show any CNV. In mice 6 weeks of age or older, every time we did laser photocoagulation we obtained CNV. The extent of the CNV was more prominent in the eyes of the one-year-old mice (group IV): 3,851 µm^2^±101.6 versus 2,930 µm^2^±205.3 in young adult group (group III), 734.9 µm^2^±79.7 in group II and 171 µm^2^±44.7 in group I. These differences were highly statistically significant between the four groups: p=0.004 between groups III and IV; p<0.0001 between groups II and III; p<0.0001 between groups I and II (Figure 6B). In this model, CNV reaction was present for several months after photocoagulation and decreased progressively. It was still present at four months for the oldest groups III and IV (2,999 µm^2^±115 for group IV versus 1,282 µm^2^±90 for group III; p<0.0001), and at eight months (2,571 µm^2^±116 in group IV versus 1,226 µm^2^±59 in group III; p<0.0001, data not shown; Figure 6A).

On flat mounts, we observed the same features. The surface of the new vessels area increased during the first two months with a stabilization observed from month 2 and a slow decrease thereafter.

**Figure 7:**
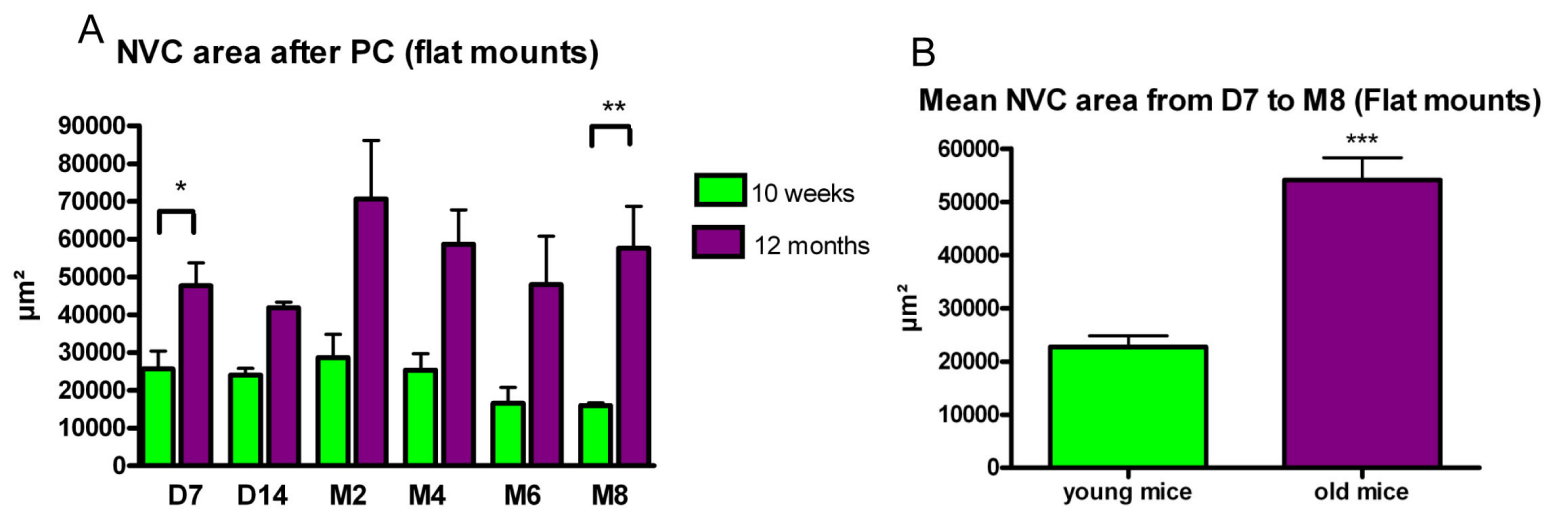
Evolution of the choroidal neovascularization reaction in young adult (group III) and old mice (group IV) as assessed on flat mounts. **A:** Choroidal neovascularization (CNV) reaction is still detected eight months after photocoagulation and is more important in 1-year-old mice during all tested time points. The difference between the two groups is significant on day 7 (p=0.028) and on month 8 (p=0.0094). **B:** The mean CNV area (during the first two weeks after photocoagulation) is larger in the 1-year-old mice; the difference is highly statistically significant according to *t*-test.

CNV area was larger in one-year-old mice (group IV) at each time point from day 7 to month 8 in comparison with the young adult mice (group III). The difference was statistically significant: p=0.028 7 days and p=0.009 eight months following photocoagulation (Figure 7A). A compilation of the data collected during the first two weeks after photocoagulation showed that the mean CNV area was consistently and significantly larger in the group IV (1-year-old mice; 45,200 µm^2^±3,490 versus 24,770 µm^2^±2,160; p<0.001) than in the group III (10–12-week-old mice; Figure 7B).

### *GFAP* mRNA expression

The hallmark of reactive gliosis is the upregulation of intermediate filament proteins such as GFAP predominantly expressed by retinal reactive astrocytes and Müller cells. Prior to laser treatment *GFAP* mRNA was expressed in the neural retinal of all mice with a significant increased expression in 1-year-old mice (p=0.025).

**Figure 8:**
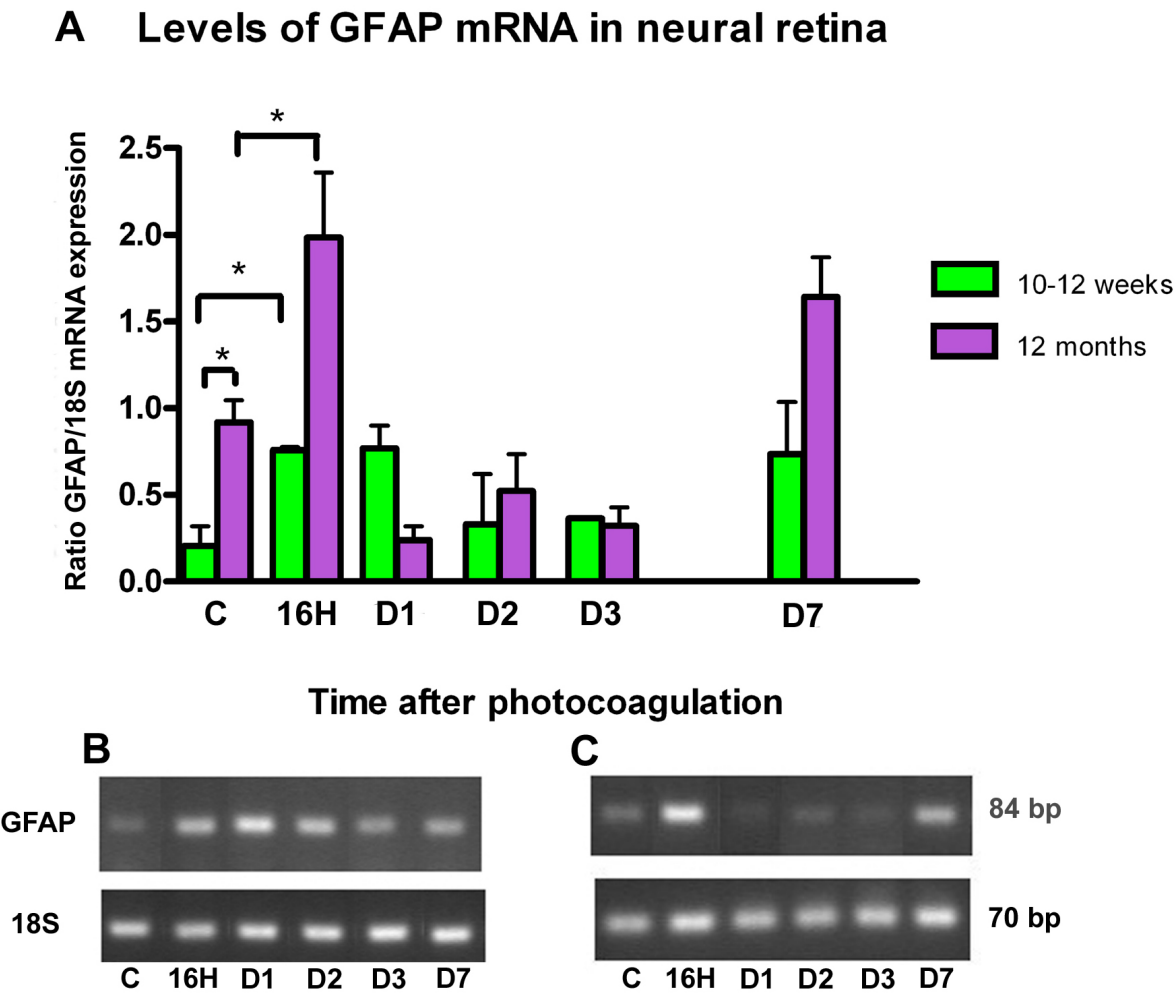
GFAP expression after photocoagulation in neural retina. **A:** The amount of glial fibrillary acidic protein (*GFAP*) transcripts was compared with *18S* transcripts (y-axis) expressed in the same samples. Four eyes were assessed at each time point. Values in histogram are means±SEM **B-C:** Representative samples of PCR fragments (individual ratio *GFAP*:*18S* closest to the corresponding mean) were analyzed by 3% agarose gel electrophoresis and visualized by ethidium bromide staining under ultraviolet light for 10–12-week-old mice (**B**) and 1-year-old mice (**C**). Prior to laser treatment, *GFAP* mRNA was highly expressed in old control mice versus young control mice (p=0.025). Following laser treatment mRNA expression was increased in the two groups, reaching higher levels at 16 h and day 7. Compared to control mice in each group, the increase of *GFAP* mRNA level is statistically significant at 16 h (p=0.04 in young group; p=0.03 in old group) and at day 7 (p=0.03 in old group).

Following laser treatment, there was a strong biphasic increase in *GFAP* expression, at 16 h-24 h and then at day7 (Figure 8). Intensity of the signal decreased thereafter, but low levels of *GFAP* mRNA remained detectable even two months after photocoagulation (data not shown).

### *VEGF* mRNA Expression

VEGF is a well known potent angiogenic factor. In untreated 10–12-week-old and 1-year-old mice, *VEGF* mRNA was expressed continuously in the RPE-choroid complex, but surprisingly it was significantly lower in one-year-old mice (group IV; p<0.001). These data are reversed however, if we consider the whole posterior part of the eye comprised of the neural retina and the RPE-choroid complex in place of the RPE-choroid complex alone. In this case VEGF mRNA was more expressed in one-year-old control mice (1.46±0.83 versus 0.87±0.30, p<0.05).

**Figure 9:**
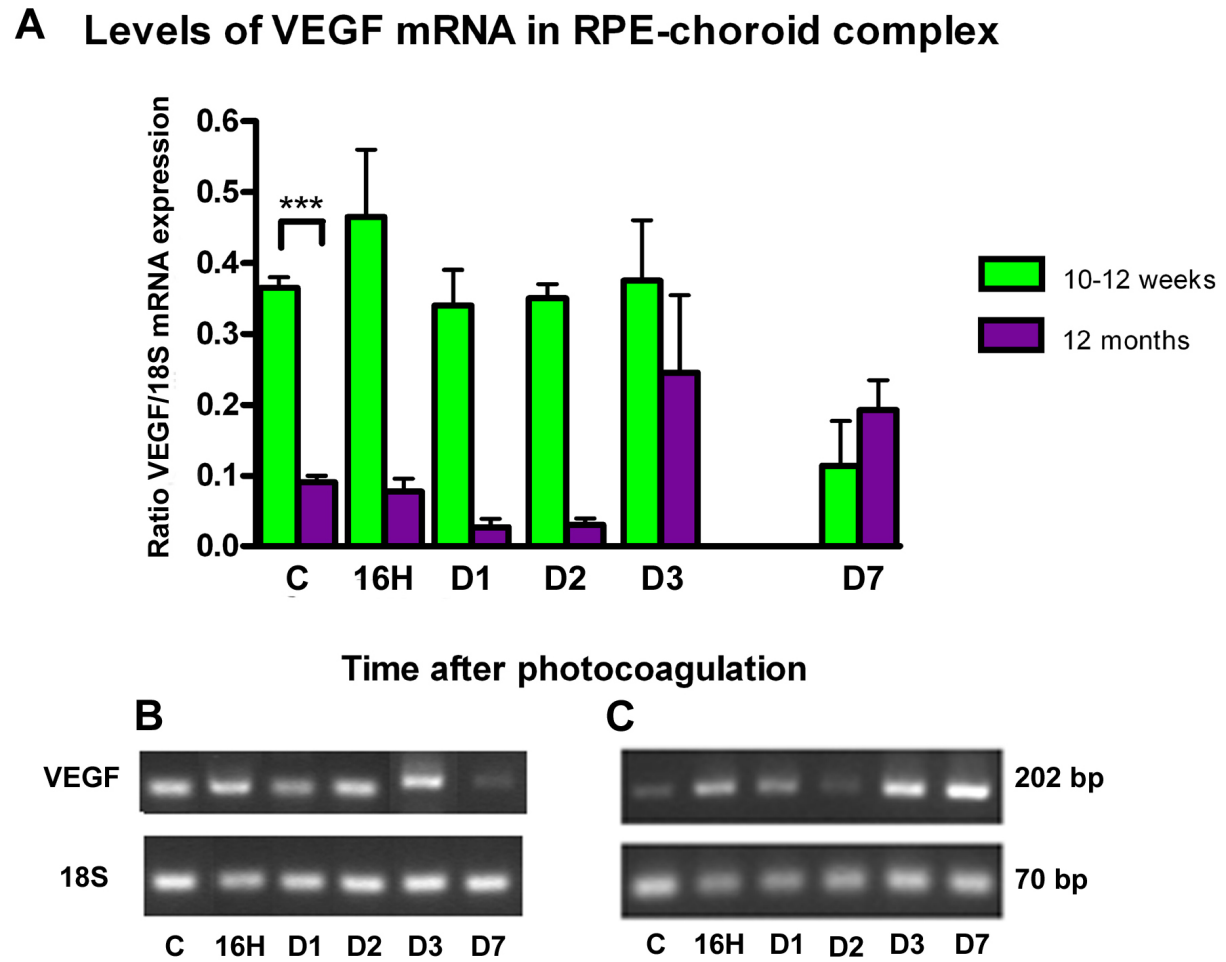
VEGF expression after photocoagulation in RPE-choroid complex. **A:** The amount of vascular endothelial growth factor (*VEGF*) transcripts was compared with *18S* transcripts (y-axis) expressed in the same samples. Four eyes were assessed at each time point. Values in histogram are means±SEM **B-C:** Representative samples of PCR fragments (individual ratio *VEGF*:*18S* closest to the corresponding mean) were analyzed by 3% agarose gel electrophoresis and visualized by ethidium bromide staining under ultraviolet light for 10–12-week-old mice (**B**) and 1-year-old mice (**C**). Prior to laser treatment, *VEGF* mRNA expression was lower in old control mice versus young control mice (p<0.001). At day 3 after PC, the highest level of *VEGF* mRNA was 2.7 times higher than the controls in old mice. In young mice the highest level of expression was reached at 16 h after photocoagulation and did not exceed 1.3 times the level observed in controls.

After photocoagulation, kinetics of *VEGF* mRNA expression was also different in 10–12-week-old mice and 1-year-old mice. In 10–12-week-old mice (group III), *VEGF* mRNA reached its peak 16 h after photocoagulation, while in one-year-old mice (group IV) the peak was observed on day 3 and declined thereafter (Figure 9). In addition, the ratio of VEGF expression in RPE-choroid complex after photocoagulation in relation to RPE-choroid complex in control not photocoagulated was more important in one-year-old mice (group IV; 2.7 times) than in 10–12-week-old mice (group III; 1.3 times).

### *MCP-1* mRNA Expression

**Figure 10:**
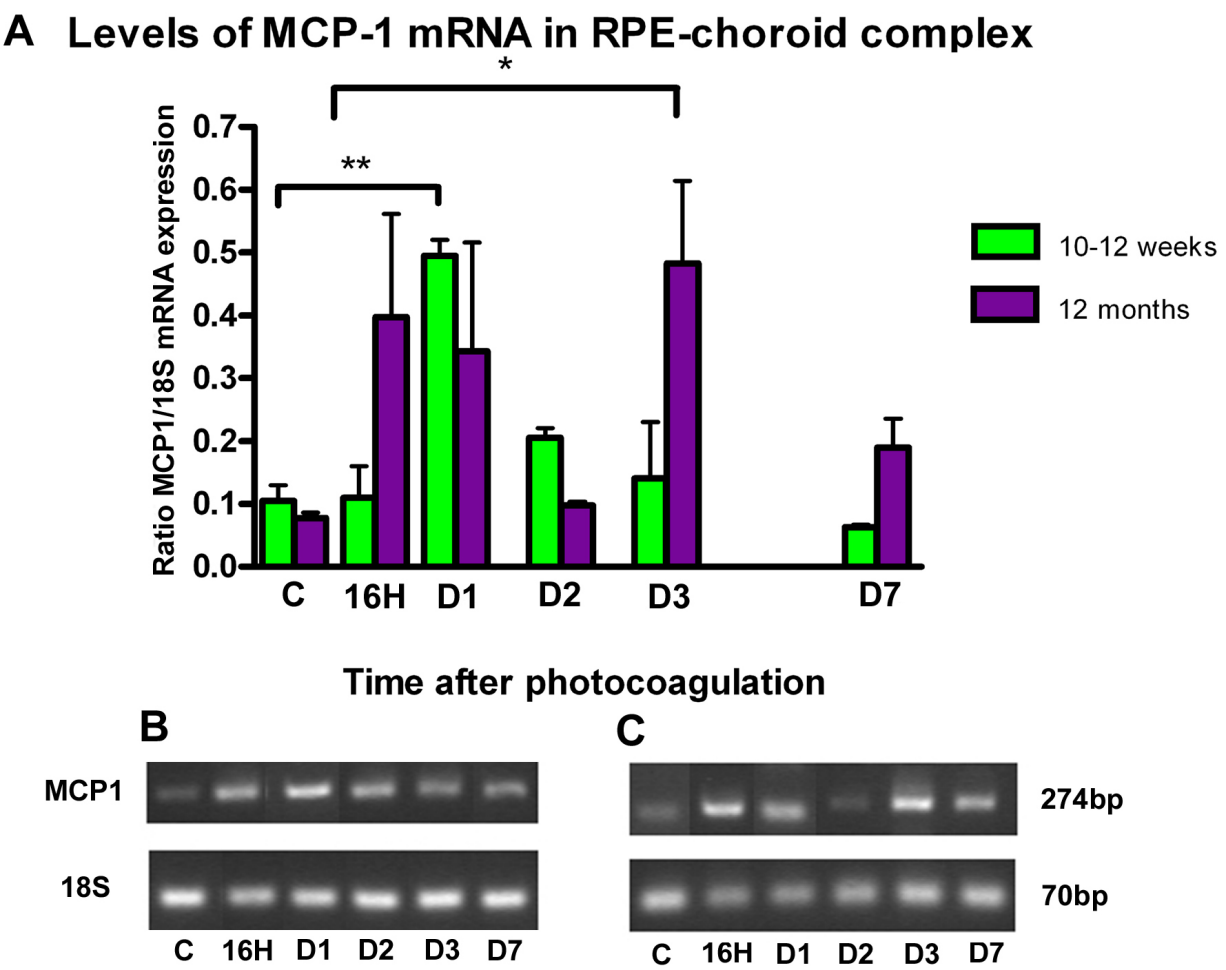
MCP-1 expression after photocoagulation in RPE-choroid complex. **A:** The amount of monocyte chemotactic protein-1 (*MCP-1*) transcripts was compared with *18S* transcripts (y-axis) expressed in the same samples. These data are from four different eyes. Values in histogram are means±SEM **B,C:** Representative samples of PCR fragments (individual ratio of MCP-1:18S closest to the corresponding mean) for 10–12-week-old mice (**B**) and 1-year-old mice (**C**) were analyzed by 3% agarose gel electrophoresis and visualized by ethidium bromide staining under ultraviolet light. Prior to laser treatment MCP-1 mRNA expression was very low. After photocoagulation, it is strongly expressed in the retinal pigment epithelium-choroid complex; its level of expression is higher and observed for a longer period of time in old mice.

*MCP-1* mRNA was slightly expressed in the RPE-choroid complex in control mice. After photocoagulation, we observed a significant increase in *MCP-1* expression at day 1 in 10–12-week-old mice (group III; p=0.008). In one-year-old mice (group IV), *MCP-1* expression reached higher levels at 16–24h and later at 3 days (p=0.02). In 1-year-old mice (group IV), *MCP-1* expression levels were higher than controls during the first week after photocoagulation (Figure 10).

## Discussion

C57Bl/6J mice of different ages demonstrate morphometric differences in the retinochoroidal wound healing processes occurring after photocoagulation. Although the same photocoagulation parameters were used in all groups of mice, the effective photocoagulation retinochoroidal lesion size assessed by the interruption of RPE and Bruch’s membrane was smaller in eyes from 1-year-old mice (group IV). This observation may be due to the thicker and less elastic Bruch’s membrane in the older mice eyes or a result of the changes in the mice eye refractive power during aging. Bruch’s membrane is an extracellular matrix composed of elastin and collagen laminae. It has been estimated that CNV invade the retinal layers through gaps in Bruch’s membrane observed during aging [2,10,11]. In humans, Bruch’s membrane demonstrates an increased thickness with age [12], and in mouse eyes [11,13,14] Bruch’s membrane loses gradually its elastic properties [11,15,16].

It is interesting that the less extensive RPE and Bruch's membrane interruption in one-year-old mice was accompanied by a more prominent CNV formation. However, the newly formed CNVs did not invade the retinal layers. Careful examination of the CNVs demonstrated that these were surrounded by pigmented cells, earlier in eyes of group IV mice than in the group III mice. A similar pigment reaction surrounding the CNV and forming tight junctions to reestablish the outer blood-retinal barrier has been previously reported [17]. It has been previously proposed that pigment encapsulation of CNV is responsible for the arrest of CNV growth and fibrosis of the lesion [18,19]. However, our observations of a persistently more extensive CNV reaction along with a more prominent pigment reaction in the eyes of the one-year-old mice of group IV do not support this hypothesis regarding the role of RPE in CNV inhibition. Therefore, we surmise that the RPE reaction and pigment encapsulation of CNV is an integral process of wound repair and healing rather than a process responsible for the arrest of CNV growth.

Data are sparse in the literature concerning aging and CNV in experimental model. To our knowledge, only one reference explored this topic, and was published by Espinosa-Heidman et al. [3] in a similar model of CNV using diode laser photocoagulation. They tested only two different age groups (2 and 16 months) and based their conclusions only on data obtained from morphological observations. They observed a more severe CNV reaction in 16-month-old mice. Our results of a more prominent CNV reaction in one-year-old mice compared to younger mice are in accord with their previous observations [3]. In the present study, we have evaluated the cellular reactions both by immunohistochemical serial sections and by flat mounting of the treated retinas, along with an extended time of follow-up. The more severe CNV reaction in 1-year-old mice was confirmed by the two techniques during all tested time points. In 4–6-week-old mice (group I and II), the resulting CNV reaction was milder and demonstrated large individual variabilities. Therefore, in our data of the long-term analysis we emphasized the results and differences observed in mice eyes from groups III and IV.

In accordance with our own results, abnormal vascular repair associated with age has been observed after blood vessel injury in several organs [20,21]. Moreover, aging has also been associated with increased vasoproliferation in a mouse model of neurodegeneration [22].

Our study provides a longitudinal observation of the CNV formation and evolution in mice of different age groups. The findings that CNV is more easily induced and results in more extensive blood vessel formation in older mice may be explained either by the higher potential induction of angiogenic factors or the depletion of inhibitory factors with age. We have previously reported that interaction between the various types of cells and the release of stimulating and inhibiting factors following photocoagulation play a crucial role in the shaping of the induced retinochoroidal wound healing and repair processes [7].

In aging, a dysregulation of the angiogenic reaction is taking place [17,23,24]. In our study, we have focused our molecular analysis on the changes occurring in gene expression of *GFAP*, *VEGF*, and *MCP*-1—three factors known to influence CNV formation following photocoagulation.We observed significant increase in *GFAP* mRNA levels in one-year-old mice compared with 10–12-week-old mice. This is consistent with data demonstrating an increase in GFAP immunolabeling of astrocytes and Müller cells in human retinas with age [25-27]. The role of glial activation on CNV remains controversial. It has been suggested that this glial reaction exerts a protective effect against oxidative stress.

By contrast, Wu et al. [27] described GFAP upregulation in human retinas with drusen and atrophic AMD and showed that activated Müller cells can release proangiogenic factors, such as VEGF, suggesting a deleterious effects of activated Müller cells on CNV. Furthermore, the inhibition of Müller cells in mice (by αAmino-Adipic-Acid intravitreal injection) diminished GFAP immunolabeling and CNV reaction [28] or after intravitreal injection of triamcinolone [8].

In the present study, we observed a marked biphasic increase in *GFAP* mRNA levels after photocoagulation in 10–12-week-old mice (group III) and one-year-old mice (group IV). One peak was observed at 16 h after photocoagulation and the second one on day 7. This suggests that the stimulus for Müller cells activation after photocoagulation is an early event that is downregulated and reactivated later at the height of new vessel formation. Humphrey et al. observed in a rat model of panretinal photocoagulation the same transient increase in *GFAP* mRNA expression but with only one peak at 24–48h [29].

Various authors have observed an increased level of *VEGF* mRNA during the first week after photocoagulation in young adult animals. Ishibashi et al. found a peak at day 3 to day 7 in a primate model. Zhao et al. [30] found a peak at day 2 to day 3 in a rat model, and Itaya et al. [31,32] observed one at day 2 in a mouse model of CNV. In all cases, *VEGF* mRNA expression decreased thereafter.

In the present study, we have observed that the level of *VEGF* mRNA expression and its kinetics are associated with the age of the treated mouse. In our experiments, we found that, in young mice (group III), the *VEGF* mRNA levels peaked at 16 h after photocoagulation. It is possible that the very early peak we observed in the 10–12-week-old mice group compared to results published in the literature is due to the difference in laser protocols used in ours and the other studies. It is of interest that while the peak expression of *VEGF* mRNA in 10–12-week-old mice reached only 1.3 times the control levels of untreated eyes, the peak expression in one-year-old mice was 2.7 times that of the controls. Thus, the signal for *VEGF* expression (and probably protein secretion) is more marked in group IV mice and may explain the more prominent CNV reaction observed.

Along with other researchers, we have previously reported that infiltrating macrophages and microglia promote CNV [8,33-35]. We have shown that the number of F4/80+ infiltrating cells reached a peak on day 3 and 7 after photocoagulation, decreasing rapidly thereafter [8]. MCP-1 is a chemokine that activates monocytes and influences the extravasation of inflammatory cells, causing monocyte accumulation [36]. *MCP-1* gene and protein are expressed by many cell types, including macrophages, endothelial cells, vascular smooth muscle cells, and epithelial cells [37]. In addition, *MCP-1* gene expression has been reported in several distinct extraocular models of inflammation and injury and in a CNV laser induced model [36,38,39]. Decreased *MCP-1* expression is associated with significant inhibition of macrophage infiltration and reduced CNV [39,40], suggesting recruitment of macrophages to the site of laser burn and the extent of neovascularization is under the control of the expression of MCP-1. Indeed both these phenomena are diminished in CCR2-deficient mice lacking the receptor for MCP-1 [33-35].

To our knowledge, the influence of aging on *MCP-1* mRNA expression in experimental CNV laser-induced models has not been previously reported. Njemini et al. [41] reported a higher level of serum MCP-1 in elderly patients. These further increased in association with inflammation and were higher than those observed in young patients with or without inflammation. In the present study, increased *MCP-1* mRNA expression in one-year-old mice may have contributed to the more prominent CNV formation recorded in older mice (group IV). Nonetheless, one cannot exclude the possibility that macrophages in old mice have different chemokine sensitivities along with cell surface receptors or signal transduction processes alterations as suggested by Ambati et al. and Njemini et al. [33,41]. In our study, we found a different profile in *MCP-1* mRNA expression in 10–12-week-old mice (group III) and one-year-old mice (group IV) after photocoagulation. These findings reinforce the possible tight association between higher macrophage infiltration along with the higher CNV formation in eyes of one-year-old mice. Moreover, since macrophages are known to produce VEGF it is possible that increased macrophages recruitment in one-year-old mice contributed to the increased VEGF expression.

Altogether, these observations confirm our previous findings [7,8] reporting the early involvement of the retinal microglia and macrophages in the wound healing processes following a laser burn in the mouse eye [42].

In conclusion, our study demonstrates that the extent of CNV formation in the photocoagulation mouse model is closely associated with the age of the treated animal. Furthermore, we observed that the retinochoroidal wound healing processes and repair after photocoagulation in the mouse eye and the more prominent gene expression of *GFAP*, *VEGF*, and *MCP-1* and their profiles play a crucial role in the resultant changes seen in the retina and choroid of the treated animal. Influence of age on the extent of CNV following phocoagulation is of interest for scientist using this model to study ocular neovascularization and angiogenesis in vivo. Moreover, these observations shed some light on the mechanisms of wound healing and repair mechanisms within the eye and may provide an insight regarding the reactions taking place during the evolution of ARMD in “younger” versus “older” patients.
